# Spatial reorganization of telomeres in long-lived quiescent cells

**DOI:** 10.1186/s13059-015-0766-2

**Published:** 2015-09-23

**Authors:** Micol Guidi, Myriam Ruault, Martial Marbouty, Isabelle Loïodice, Axel Cournac, Cyrille Billaudeau, Antoine Hocher, Julien Mozziconacci, Romain Koszul, Angela Taddei

**Affiliations:** Institut Curie, PSL Research University, Paris, F-75248 France; CNRS, UMR 3664, Paris, F-75248 France; Institut Pasteur, Department Genomes and Genetics, Groupe Régulation Spatiale des Génomes, 75015 Paris, France; CNRS, UMR 3525, 75015 Paris, France; LPTMC, Université Pierre et Marie Curie, UMR 7600, Sorbonne Universités, 4 Place Jussieu, 75005 Paris, France; Sorbonne Universités, UPMC Univ, Paris 06, France

## Abstract

**Background:**

The spatiotemporal behavior of chromatin is an important control mechanism of genomic function. Studies in *Saccharomyces cerevisiae* have broadly contributed to demonstrate the functional importance of nuclear organization. Although in the wild yeast survival depends on their ability to withstand adverse conditions, most of these studies were conducted on cells undergoing exponential growth. In these conditions, as in most eukaryotic cells, silent chromatin that is mainly found at the 32 telomeres accumulates at the nuclear envelope, forming three to five foci.

**Results:**

Here, combining live microscopy, DNA FISH and chromosome conformation capture (HiC) techniques, we report that chromosomes adopt distinct organizations according to the metabolic status of the cell. In particular, following carbon source exhaustion the genome of long-lived quiescent cells undergoes a major spatial re-organization driven by the grouping of telomeres into a unique focus or hypercluster localized in the center of the nucleus. This change in genome conformation is specific to quiescent cells able to sustain long-term viability. We further show that reactive oxygen species produced by mitochondrial activity during respiration commit the cell to form a hypercluster upon starvation. Importantly, deleting the gene encoding telomere associated silencing factor *SIR3* abolishes telomere grouping and decreases longevity, a defect that is rescued by expressing a silencing defective *SIR3* allele competent for hypercluster formation.

**Conclusions:**

Our data show that mitochondrial activity primes cells to group their telomeres into a hypercluster upon starvation, reshaping the genome architecture into a conformation that may contribute to maintain longevity of quiescent cells.

**Electronic supplementary material:**

The online version of this article (doi:10.1186/s13059-015-0766-2) contains supplementary material, which is available to authorized users.

## Background

The spatiotemporal behavior of genomes and their regulatory proteins is an important control mechanism of genomic function. One of the most pervasive features of nuclear organization is the existence of subnuclear compartments, which are thought to create microenvironments that favor or impede specific DNA- or RNA-related processes [1]. Deciphering how the dynamics of this subnuclear compartmentalization are regulated in relation to changes in genome activity is a key step in understanding how nuclear organization participates in nuclear function.

Well-characterized examples of subnuclear compartments include clusters of specific genes or repetitive DNA sequences [2], such as telomeric repeats (in budding yeast) or centromeric satellites (in fission yeast, fly and mammals) and retrotransposons (in fission yeast, Tn2/Ku70-mediated clustering) [3]. These repetitive sequences generally nucleate patterns of histone modifications that are recognized by histone-binding repressors, and their clustering results in the sequestration of these general repressors into subcompartments. Besides its role in concentrating silencing factors, this evolutionarily conserved phenomenon has a dominant impact on chromosome folding and positioning. In metazoans, a cell type-specific nuclear distribution of heterochromatin is established upon cell differentiation, and is often compromised in cancer cells [4]. In budding yeast, the clustering of silent chromatin provides an excellent model of a subnuclear compartment.

Most *Saccharomyces cerevisiae* functional and structural studies have been conducted on exponentially growing cell cultures. In these conditions, silent chromatin is mainly found at telomeres and at the cryptic mating type loci (*HM* loci), where it is generated by the recruitment of the SIR complex comprising Sir2, Sir3, and Sir4. At telomeres, this nucleation event is achieved by the transcription factor Rap1, which binds the telomere TG repeats and interacts with Sir3 and Sir4. Sir4 heterodimerizes with the NAD + −dependent histone deacetylase Sir2, which deacetylates H4 histone tails from neighboring nucleosomes, thus generating binding sites for Sir3. The SIR complex thus spreads over a 2–3-kb subtelomeric region leading to the transcriptional repression of subtelomeric regions.

The clustering of telomeres into perinuclear foci generates a zone that favors SIR-mediated repression at the nuclear periphery [5, 6] and ensures that SIR proteins do not bind promiscuously to repress other sites in the genome [7, 8]. Furthermore, telomere anchorage in S phase contributes to proper telomerase control and suppresses recombination among telomere repeats [9, 10].

The average large-scale organization of budding yeast chromosomes during exponential growth has been described through genome-wide capture of chromosome conformation (3C) experiments [11]. This analysis unveiled a polarized configuration with chromosome arms extending away from the centromeres that are held by the spindle-pole body, in agreement with microscopy data [12]. This so called Rabl organization — initially observed by Carl Rabl in rapidly dividing nuclei of salamanders [13] — can be mimicked to some extent by polymer models using a limited number of assumptions [11, 14–16]. However, it remains unclear how specific biological processes could affect this robust average organization.

As mentioned above, most of the studies characterizing genome organization and its functional consequences in budding yeast have been conducted in nutrient-replete conditions with cells undergoing exponential growth. However, yeast cells rarely experience such a lush environment and their survival in the wild depends on their ability to withstand adverse conditions.

**Fig. 1:**
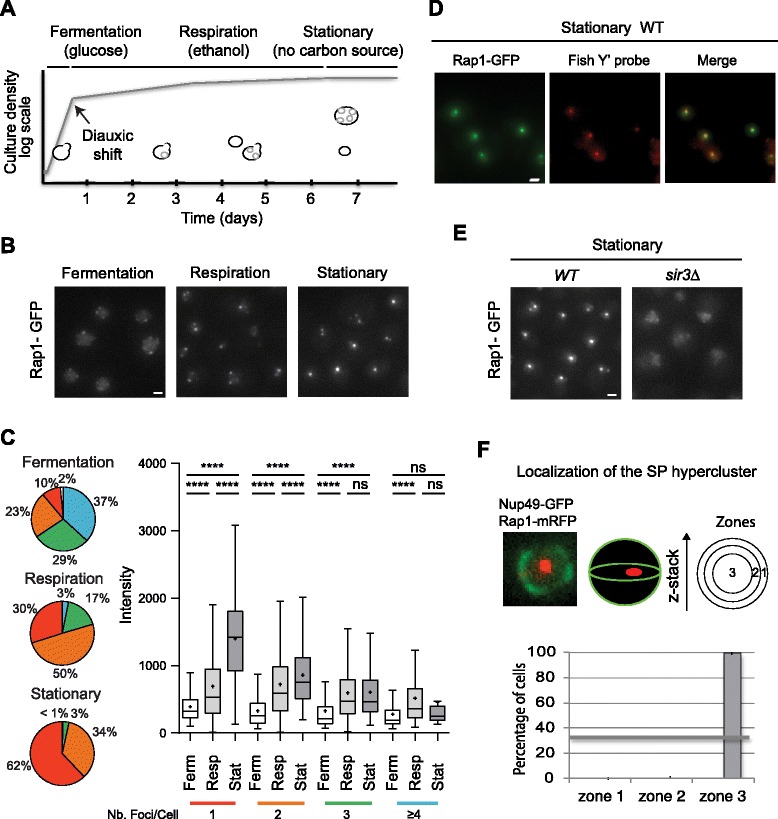
Massive telomere reorganization upon carbon source exhaustion. **a** Growth curve for *S. cerevisiae* grown in rich glucose-based liquid medium. Yeast cells grown in medium containing glucose divide exponentially, mainly perform glycolysis, and release ethanol into the medium. When glucose becomes limiting (roughly after 12 hours in the conditions used in this study; see “Materials and methods”) the cells undergo a major metabolic transition called “diauxic shift”, during which they stop fermentation and start aerobic utilization of ethanol (respiration phase). After this transition, cells divide slowly and become more resistant to different stresses. Once ethanol is exhausted and no other carbon source is available, around 7 days, the culture enters the stationary phase (*SP*). At this stage, the majority of the cells are in a quiescent state. **b** Representative fluorescent images of the telomere-associated protein Rap1 tagged with green fluorescent protein (*GFP*). Overnight wild-type (*WT*) “yAT1684” liquid cultures were diluted to 0.2 OD_600nm_/ml and images were acquired after 5 hours (1 OD_600nm_/ml, fermentation phase), 2 days (28 OD_600nm_/ml, respiration phase) and 7 days (40 OD_600nm_/ml, stationary phase). **c** Quantification of the distribution of intensity and number of foci of Rap1-GFP images from experiment shown in (**b**) with our in-house software. Pie charts represent percentages of cells with 0 (*white*), 1 (*red*), 2 (*orange*), 3 (*green*) and 4 (*blue*) foci. Box plots: *white* = fermentation (*Ferm*), *light gray* = respiration (*Resp*), *dark grey* = stationary (*Stat*). Median (*line*) and mean (*cross*) are indicated. For each condition, more than 1000 cells were analyzed. Statistical tests were carried out using the Mann–Whitney non-parametric test (****p < 0.0001; ***p < 0.001; **0.001 < *p* < 0.01; *0.01 < *p* < 0.05; *ns* = *p* > 0). **d** Colocalization of telomeres with Rap1 foci. ImmunoFISH with Y’ probes was performed on a WT strain yAT1684 at SP. **e** Representative fluorescent images of the telomere-associated protein Rap1 tagged with GFP in SP WT and *sir3*∆ cells. **f** Rap1-GFP hypercluster localization relative to the nuclear pore. Two-color z-stack images were acquired on a WT strain yAT2407 expressing Rap1-yemRFP and the GFP tagged nucleoporin 49 (Nup49-GFP) during SP. The localization of the Rap1-yemRFP hypercluster in one of the three equal concentric zones of the nucleus was scored in the focal plane. This experiment was repeated twice and for each experiment >100 nuclei with a hyper-cluster were analyzed

It is well known that yeast cells finely tune their growth and behavior to their environment, adapting to nutritional depletion or stresses by engaging specific developmental programs [17]. When grown in rich media containing glucose, they progress through distinct metabolic programs (Fig. 1a), with each transition being accompanied by widespread transcriptional reprogramming [18, 19]. In the first phase (exponential phase), cells metabolize glucose predominantly by glycolysis, releasing ethanol in the medium. When glucose becomes limiting, the culture enters diauxic shift, a transition characterized by a decreased growth rate and a metabolic switch from glycolysis to aerobic utilization of ethanol. Finally, when no other carbon source is available cells enter stationary phase (SP). During that stage most cells are in quiescence, a non-proliferative state that maintains the ability to resume growth following restoration of missing nutrients.

Recent studies in different species demonstrated that a hostile environment (i.e., caloric restriction or the presence of mild oxygen stresses) can trigger a “vaccination-like” adaptive response leading to the acquisition of anti-aging functions [20]. Following the same principle, budding yeast can reach different quiescent states depending on the conditions that induce the cell cycle exit, each of them leading to different outcome in terms of chronological lifespan (CLS) [21]. Deciphering the key features that differentiate each metabolic state is essential to understand mechanisms that extend lifespan in yeast.

Here we show that, following carbon source exhaustion, the silencing factor Sir3 drives the telomeres of quiescent cells to group together, forming a discrete, large cluster (hypercluster) at the center of the nucleus. This organization is specific to quiescent cells able to sustain long-term viability. Our data strongly support a model in which mitochondrial activity, through the production of reactive oxygen species (ROS) during cell respiration, commits cells to form a telomere hypercluster upon starvation. Importantly, *sir3*∆ cultures, which are defective in forming telomere hyperclusters in SP, show reduced CLS. Furthermore, expressing a silencing-defective *SIR3* allele rescues both telomere distribution and the CLS of a *sir3* null strain, strongly arguing that telomere clustering directly contributes to cell survival during quiescence.

## Results

### Massive telomere reorganization upon carbon source exhaustion

To investigate telomere organization in live cells, we monitored the subnuclear distribution of the telomeric protein Rap1 fused to green fluorescent protein (GFP) [22] at different stages of a liquid culture, from glycolysis to late respiration to SP. We observed dramatic changes in the distribution of the Rap1-GFP signal during this time course (Fig. 1a, b). In agreement with previous reports [6, 22], Rap1-GFP formed three to five foci during the logarithmic phase, quantified using our custom-made software (Fig. 1c; adapted from [22]). In cells undergoing respiration (after 2 days in culture), Rap1-GFP foci were fewer and brighter, with 50 % of the cells showing two foci and 30 % of the cells having only one focus (versus 23 % and 10 %, respectively, during fermentation). In SP 62 % of the cells exhibited a unique focus with a median intensity that was fivefold higher than in the exponential phase. Moreover, we noticed that when the number of foci per cell decreases, the intensities of the remaining foci increase (Fig. 1c), suggesting that smaller foci group into larger ones. Importantly, we verified that the brightness of the Rap1-GFP clusters observed in SP was not due to an overall increase in Rap1-GFP levels (Additional file 1: Figure S1a). Furthermore, we observed a similar clustering with SIR complex proteins fused to GFP (Sir2/3/4; Additional file 1: Figure S1b). We confirmed that Rap1-GFP foci coincided with the Y’ telomeric clusters and Sir3 foci in SP cells by combined immunostaining and fluorescence in situ hybridization (immuno-FISH; Fig. 1d) and in vivo imaging (Additional file 1: Figure S1c). Thus, telomere-associated silent chromatin groups into “hyperclusters” in SP cells.

As in exponentially growing cells, telomere hyperclustering requires *SIR3* and *SIR4* in SP cells (Fig. 1e; Additional file 1: Figure S1d). Although the brightest Rap1-GFP focus was mainly found adjacent to the nuclear envelope in exponentially growing cells [6, 22], telomere hyperclusters were overwhelmingly found in the innermost area in SP cells (>90 % in zone 3; Fig. 1f).

We next evaluated whether other nuclear landmarks were also altered in SP. In agreement with previous reports, we found that the nuclear diameter (data not shown, inferred from experiment Fig. 1f) was smaller and the nucleolus more compact in cells after the diauxic shift (Additional file 1: Figure S1e) [23]. Consistent with [24], we noticed that kinetochore proteins formed a “bundle” in a subpopulation of cells; however, this structure did not correlate with telomere hyperclusters (Additional file 1: Figure S1f). Furthermore, we did not observe major changes in the distribution of the centromere-associated protein Cse4 in SP cells containing telomere hyperclusters (Additional file 1: Figure S1g). Thus, a specific SIR-dependent re-organization of telomeres occurs in a subpopulation of SP cells.

### Hyperclustering of telomeres occurs only in the long-lived fraction of SP cells

**Fig. 2:**
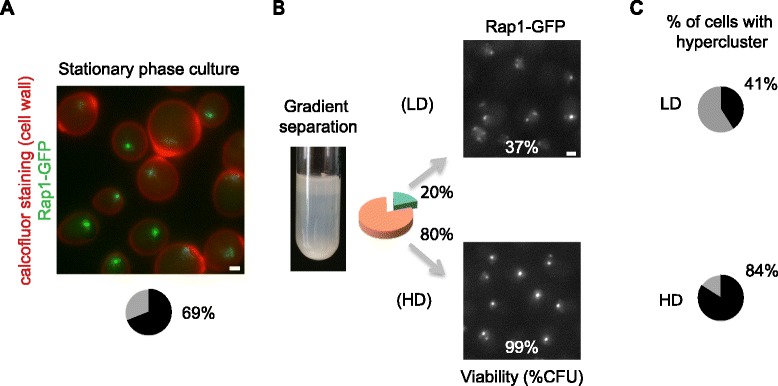
Telomeres hypercluster in the long-lived fraction of stationary phase cells. **a** Representative image of wild-type strain yAT1684 in SP: telomeres are visualized through Rap1-GFP and bud scars within the cell wall are stained with Calcofluor-white. **b** Picture of a Percoll gradient separation tube of a wild-type yAT1684 SP culture. *HD* high density, *LD* low density. The pie chart represents the distribution of LD (*green*) and HD (*orange*) cells within the population. Representative fluorescent images of Rap1 tagged with GFP of the LD and HD cell fractions are shown on the right. Percentages indicate the colony-forming ability of the two fractions measured as percentage of colony-forming units (*CFU*). **c** Quantification of the distribution of intensity and number of foci of Rap1-GFP images from experiment shown in (**b**) with our in-house software. Pie charts represent the percentage of cells with telomere hyperclusters within the population (*black*)

As previously reported [25], SP cultures consist of different types of cells. Equilibrium density-gradient centrifugation enables the separation of a dense fraction mainly composed of small unbudded daughter cells that are able to sustain long-term viability, and a lighter fraction that includes both budded and unbudded cells that rapidly lose the ability to perpetuate over time. Calcofluor staining revealed that cells with hyperclusters (defined as cells containing one or two foci and at least one Rap1-GFP focus with intensity levels above 95 % of foci in exponentially growing cells) are essentially small unbudded cells (Fig. 2a). Sorting SP cells by density gradient enriched the population of cells showing hyperclusters from 69 % to 84 % in the densest fraction (HD) while most cells from the less dense fraction (LD) showed a distribution of Rap1-GFP foci similar to the post-diauxic shift cells (Figs. 1b, c and 2b, c). Moreover, we confirmed that the viability is significantly lower for the lighter cells than for the denser ones that show hyperclusters (37 % versus 99 %, respectively). We thus conclude that telomere hyperclustering occurs specifically in quiescent SP cells.

### The global chromosome organization in long-lived SP cells is constrained by centromere and telomere clustering

**Fig. 3:**
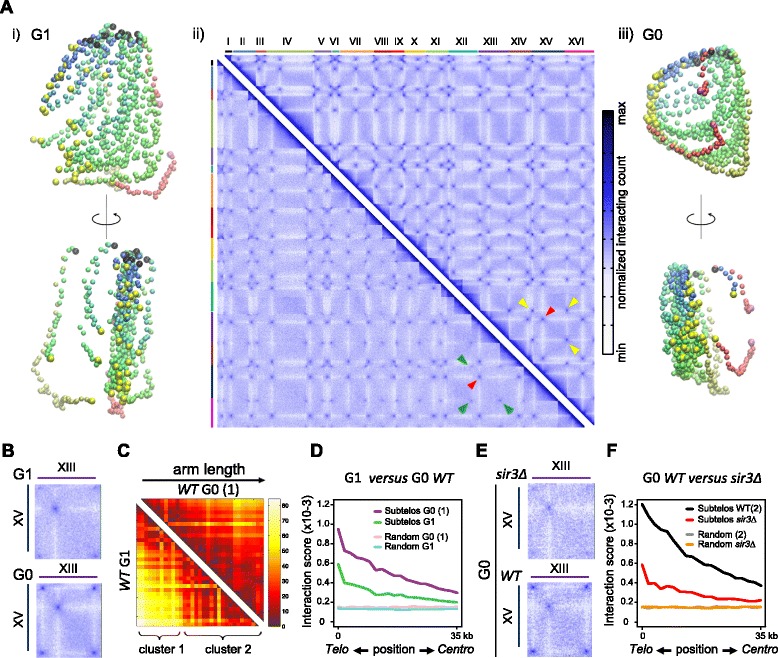
Sir3-dependent hyperclustering of telomeres is the prominent feature of genome folding in long-lived quiescent SP cells. **a** Chromosome organization of G1 and quiescent cells (HD fraction of SP: G0). *ii*) Normalized genomic contact matrix obtained for G1 daughter (*left*) and quiescent (*right*) cells. The chromosome names are indicated on the top axis. The color scale on the right indicates the frequency of contacts between two regions of the genome (*white* = rare contacts, *dark blue* = frequent contacts). *Red arrowheads* indicate centromere clustering; *green* and *yellow arrowheads* point at telomere–telomere contacts between two chromosomes (*XIII* and *XV*) in G1 and G0 cells, respectively. The average 3D structures reconstructed from the two contact maps are depicted on the corresponding side (see also Additional files 2 and 4). Each chromosome is represented as a chain of beads (1 bead = 20 kb), and the color code reflects the chromosome arm length, from *blue* for short arms to *red* for long arms. *Yellow beads* = subtelomeric regions; *black beads* = centromeres; *purple beads* = boundaries of the rDNA cluster on chromosome 12. **b** Scaled up view of a region of the matrices corresponding to the contacts between chromosomes XV and XIII in the G0 and G1 stages. **c** Representation of the distances between all pairs of telomeres as observed in the 3D structures of G1 and quiescent cells. Both structures have been scaled to account for the measured difference in size between nuclei in G0 and G1 daughter cells (unit=10nm, see “Materials and methods”). The 32 telomeres are ordered according to the corresponding chromosome arm length, from the shortest (*left*) to the largest (*right*). *WT* wild type. **d** Analysis of the contact frequency between sub-telomeres in G1 and G0 quiescent cells. For 3-kb windows starting at the telomere (*right*) and moving toward the centromeres, the mean of contact from each window with the other subtelomeres is plotted. The *blue* and *pink curves* represent the contacts computed between 35-kb segments randomly sampled from the genome in both conditions, to illustrate the absence of coverage bias after normalization in the analysis. **e** Scaled up view of the contacts between chromosomes XV and XIII in the G0 stage in *SIR3* defective (*sir3*∆, *hml*∆ to avoid the pseudo-diploid effect due to *SIR3* deletion) or WT (*hml*∆) cells (see Additional file 3 for a genome-wide overview of the contacts in these experiments). **f** As in (**d**) for *sir3*∆ and WT G0 cells

To decipher the three-dimensional (3D) organization of the entire genome in long-lived SP cells, we turned to 3C [26]. We used an untagged strain to avoid any possible artifact related to the expression of tagged telomere proteins. As cells from the dense fraction of SP are small unbudded cells (Fig. 2a), we compared the genomic contact maps of these cells with G1 daughter cells elutriated from an exponential culture to avoid the contribution of the cell cycle in this latter case. In order to facilitate the interpretation of the contact map [Fig. 3a(ii)], the matrix was converted into a 3D map in which the distance between each pair of genome segments was optimized to reach the inverse of their measured contact frequency [Fig. 3a(i); Additional file 2) [27]. This 3D reconstruction of the entire contact map provided a remarkable overview of the average yeast genome organization in a population of G1 cells, with the rDNA clearly isolated from the rest of the genome, a dense centromere cluster, and a tendency for subtelomeres to co-localize, consistent with the well-documented perinuclear clustering of telomeres [6].

In excellent agreement with our microscopy data, contacts between telomeres became prominent in quiescent cells, generating a remarkable hypercluster. The influence of chromosome arm length on the subtelomere contacts — which in exponentially growing cells discriminates two groups of telomeres exhibiting preferential contacts with each other’s — is alleviated by the formation of the hypercluster, suggesting the formation of a grid-like/homogeneous disposition of telomeres (Fig. 3b, c) [11, 28]. In addition, regions closer to the telomeres exhibited an increased number of contacts in SP, whereas the number of contacts between centromeres decreased slightly (Fig. 3d; Additional file 3: Figure S2a). Thus, the frequency of contacts increases specifically between telomeres, imposing a general constraint on the whole genome organization, with each chromosome arm now being tethered at two points of the nuclear space (Fig. 3a(iii); Additional file 4). As a result, the average contacts between chromosome arms, which are primarily constrained by their sizes and centromere clustering in G1 (Fig. 3a(i), c), appear distorted due to subtelomere interactions in G0 (Fig. 3a(iii), d). Importantly, these observations were confirmed in two different genetic backgrounds (BY and W303; Additional file 3: Figure S2c, d).

To test whether this reorganization is driven by increased telomere–telomere interactions, we compared the genomic contact map of cells in which *SIR3* had been deleted and wild-type cells from the dense fraction of a SP culture. In agreement with our microscopy data (Fig. 1e) we observed that *sir3*∆ cells were not able to generate a hypercluster upon entry into quiescence (Fig. 3e, f; Additional file 3: Figure S2b) and that the general organization of chromosomes in *sir3*∆ quiescent cells resembles the organization of wild-type G1 cells, with similar levels of contacts between subtelomeric regions (Fig. 3d, f; Additional file 3: Figure S2b). We thus conclude that the main changes in chromosome organization that occur as cells enter quiescence are driven by an increase in Sir3-dependent telomere clustering.

### Telomeres form hyperclusters specifically in conditions inducing long-lived quiescent cells

**Fig. 4:**
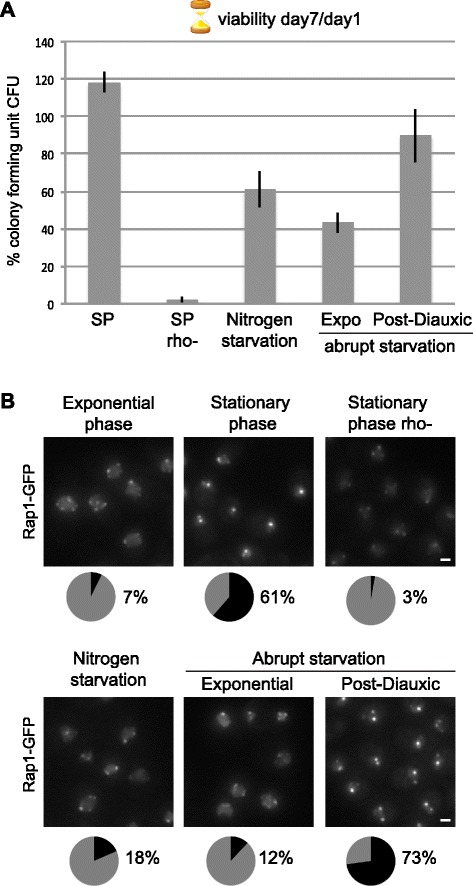
Telomere hyperclusters are a feature of long-lived quiescent cells and require mitochondrial activity. **a** Colony forming ability measured as percentage of colony forming units (*CFU*) of WT strain yAT1684 after 7 days in quiescence induced by different methods: carbon exhaustion from YPD (*SP*); SP respiratory-deficient cells (*SP rho-*); nitrogen starvation; abrupt starvation of exponential and post-diauxic cells. Cells were plated at day 1 and day 7 after quiescence induction and the ratio day 7/day 1 was considered as the day 7 CLS. Standard deviations from three experiments are indicated. **b** Representative fluorescent Rap1-GFP images of cultures used for the CFU assay shown in (**a**). Cells were imaged at day 1 CLS. Pie charts represent the percentage of cells with telomere hyperclusters within the population (*black*)

To test whether telomere hyperclusters were a general feature of quiescence we compared telomere subnuclear distribution in quiescent cells induced by different means. As mentioned above, although quiescent cells are by definition viable, their CLS properties depend on the method/metabolic changes used to induce the cell cycle exit [21] (Fig. 4a). At day 7 of CLS 61 % of quiescent cells arising from progressive carbon source exhaustion (SP) had formed telomere hyperclusters and these retained >90 % viability (Fig. 4a, b). In contrast, quiescent cells induced by nitrogen starvation formed hyperclusters at a much lower rate (18 % had done so) and lost viability more rapidly, as previously reported [21]. Hence, the grouping of telomere foci into hyperclusters is not a consequence of cell cycle arrest but rather a specific feature of long-lived quiescent cells induced by carbon source exhaustion.

### The ability to form telomere hyperclusters upon starvation is acquired during respiration

Interestingly, when abruptly starved from carbon source, cells respond differently depending on their initial metabolic status: few cells previously undergoing glucose fermentation formed telomere hyperclusters upon starvation (7 %) and showed a strong decrease in viability at day 7 (≈40 %), in agreement with previous reports [21, 29]. In contrast, 73 % of cells previously undergoing respiration (post-diauxic shift) formed telomere hyperclusters upon starvation and these retained ≈ 90 % of viability at day 7. Thus, only cells that experienced respiration before entering quiescence had a long CLS (>90 % viability after 1 week of starvation) and formed telomere hyperclusters at rates of more than 60 % (Fig. 4a, b). These characteristics could be attributed either to their metabolic activity or to their growth rates, as cells undergoing respiration divide slower and slow growth confers resistance to various stresses [30]. However, slow growth was not sufficient to prime cells to form a hypercluster upon starvation, as cells grown slowly in glucose at 25 °C and starved after fermentation did not form hyperclusters (Additional file 5). To determine if respiration was an obligatory step to induce telomere hyperclustering upon starvation, we monitored telomere clustering in respiratory deficient cells (rho-) after glucose exhaustion (Fig. 4b) or upon abrupt starvation (data not shown). These conditions led to a very low rate of cells with bright Rap1-GFP foci (3 %; Fig. 4b) indicating that respiration, or at least mitochondrial metabolism, favors the formation of telomere hyperclusters upon abrupt starvation. It is noteworthy that rho- cells show very short chronological lifespan in SP (Fig. 4a), consistent with our observation that telomere hyperclusters are a feature of long-lived quiescent cells. These data indicate that the ability to form hyperclusters is favored by mitochondrial activity.

### Hormetic ROS during exponential phase prime cells to form hyperclusters upon starvation and to sustain long-term viability

**Fig. 5:**
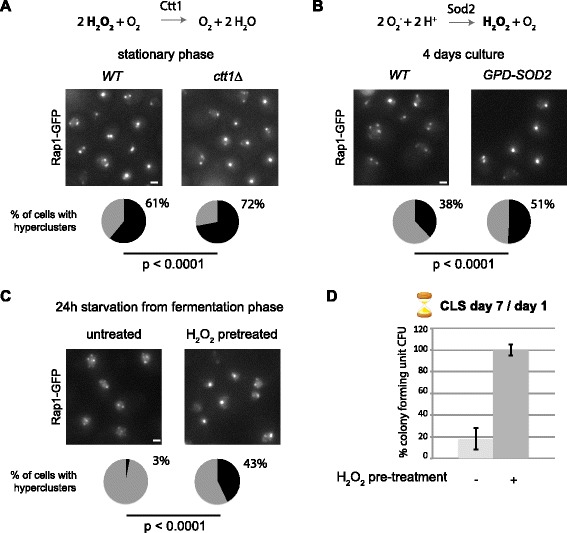
H_2_O_2_ signal during cell growth primes cells to sustain long-term viability and to form hyperclusters upon starvation. **a** *Top*: summary scheme of Ctt1 catalase activity. *Center*: Rap1-GFP representative images of SP wild-type (*WT*) and *ctt1*∆ cultures. Quantification of the distribution of intensity and number of foci of Rap1-GFP images was performed with our in-house software. Pie charts at the bottom represent the percentage of cells with telomere hyperclusters (*black*) within the population. **b** *Top*: summary scheme of Sod2 superoxidase activity. *Center*: Rap1-GFP representative images of WT and *GPD-SOD2* cultures at 4 days in YPD (late respiration). Quantification of the distribution of intensity and number of foci of Rap1-GFP images was performed as in (**a**). **c** The effect of H_2_O_2_ (1 mM) on hyperclustering commitment. WT yAT1684 cells undergoing fermentation with or without H_2_O_2_ treatment were starved for 16 h in water and then imaged. Representative fluorescent Rap1-GFP pictures are shown. Pie charts represent the percentage of cells with telomere hyperclusters (*black*) within the population. For each condition, more than 1000 cells were analyzed. Statistical tests were carried out using a two-proportion Z test. **d** Colony forming ability measured as percentage of colony forming units (*CFU*) of the cultures from (**c**) after 7 days of starvation. Cultures were plated at day 1 and day 7 of starvation and the ratio day 7/day 1 is reported. Standard deviations from three experiments are indicated

We reasoned that ROS, as byproducts of the respiration process, could prime cells to form telomere hyperclusters upon starvation. Indeed, studies in model organisms show that a mild increase in ROS levels can positively influence health and lifespan, a process defined as mitochondrial hormesis or mitohormesis [20, 31]. Since hydrogen peroxide (H_2_O_2_) has emerged as a ROS signaling molecule able to induce an adaptive response [32], we tested the effect of increasing intracellular H_2_O_2_ on telomere hypercluster formation. This was achieved either by deleting the gene encoding the cytoplasmic catalase Ctt1, which scavenges H_2_O_2_ [33], or by overexpressing the superoxide dismutase Sod2, which converts O_2_- into H_2_O_2_ (Fig. 5a, b). In agreement with our hypothesis, we observed that telomere hyperclusters formed more efficiently in SP of *ctt1*∆ cells, and appeared earlier in cells overexpressing *SOD2*, compared with wild-type cells (Fig. 5a, b). Importantly, these strains deleted for *CTT1* or overexpressing *SOD2* both show extended lifespan [33, 34].

We next tested whether increasing ROS levels in fermenting cells by treating them with H_2_O_2_ would bypass the requirement for the respiration phase and promote hypercluster formation upon starvation. As expected, untreated cells were unable to form telomere hyperclusters after starvation (Fig. 5c) and had a short CLS (Fig. 5d). In contrast, H_2_O_2_ pre-treated cells contained brighter and fewer Rap1-GFP foci (Fig. 5c). Importantly, like SP HD cells, H_2_O_2_ pre-treated cells had >90 % viability at day 7 of CLS (Fig. 5d). Combined, these data strongly suggest that ROS exposure prior to starvation promotes telomere grouping and long-term viability during starvation.

### Sir3-dependent telomere clustering favors long term survival during quiescence

We previously demonstrated that telomere grouping in exponentially growing cells is dependent on Sir3 protein amount but independent of silencing [22]. We found that telomere hyperclustering in wild-type quiescent cells is not driven by an increase in Sir3 protein levels as revealed by western blot analysis (Additional file 6: Figure S4a). Furthermore, monitoring Sir3 occupancy genome-wide by chromatin immunoprecipitation (ChIP) revealed no significant changes in Sir3 spreading between exponentially growing cells and SP cells showing telomere hyperclusters (Additional file 6: Figure S4b).

**Fig. 6:**
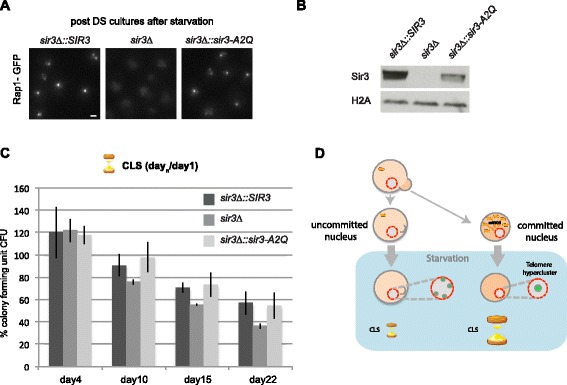
Sir3 dependent telomere clustering contributes to sustain long chronological lifespan. **a** Rap1-GFP representative images of *sir3*∆*::SIR3* “yAT2332”, *sir3*∆ “yAT2338” and *sir3∆* *::sir3-A2Q* “yAT2333” grown 3 days in YPD and starved overnight in water. *DS* diauxic shift. **b** Western blot against Sir3 and H2A on crude extracts from SP cultures of *sir3*∆*::SIR3* “yAT2332”, *sir3*∆ “yAT2338” and *sir3*∆*::sir3-A2Q* “yAT2333”. **c** CFU assay on *sir3*∆*::SIR3* “yAT2332”, *sir3*∆ “yAT2338” and *sir3*∆*::sir3-A2Q* “yAT2333”. Cells were grown in 3 days in YPD, transferred in water and plated at day 1 (**a**), day 10, day 15, and day 22. The ratios day 4/day 1, day 10/day 1, day 15/day 1, and day 22/day 1 are shown. **d** Summary scheme of long-lived quiescent cells showing a programmed reorganization of silent chromatin triggered by mitochondrial activity. Telomeres are organized in three to four foci localized at the nuclear periphery during fermentation. After the diauxic shift, ROS coming from the mitochondria commit cell nuclei to form telomere hyperclusters during starvation and to sustain long CLS. On the other hand, mother cells that are not committed to telomere hyperclustering will rapidly lose viability during starvation

To evaluate whether the silencing function of Sir3 is required for telomere hyperclustering and for longevity in quiescent cells, we transformed *sir3*Δ cells (defective for telomere clustering) with either a wild-type or a silencing dead copy of *SIR3* (*sir3-A2Q*) [22] and assessed their CLS. We found that the insertion of either *SIR3* or *sir3-A2Q* rescued the telomere hyperclustering in quiescent cells (Fig. 6a). We noticed that Rap1-GFP foci in the *sir3-A2Q* mutant were dimmer than in the *SIR3* strain, probably due to a lower stability of this mutant form of Sir3 in SP (Fig. 6b). Nevertheless, this establishes that the silencing function of Sir3 is not required for telomere clustering in quiescence.

The *sir3*∆ strain had viability similar to wild-type cells at days 4 and 7 (Fig. 6c and not shown), arguing that this mutant enters properly into quiescence upon carbon source exhaustion. In agreement with this, we observed that the *sir3*∆ strain generates dense cells following the diauxic shift. Furthermore, these cells are as thermo-tolerant as their wild-type counterpart (Additional file 6: Figure S4c–e). In contrast, the *sir3*∆ strain shows a modest decrease in viability after day 10 compared with wild type, suggesting that while Sir3 is dispensable to enter into the quiescent state, it contributes to the maintenance of this specific cellular state. Importantly, expressing the *sir3-A2Q* mutant rescued the viability defect observed in the *sir3*∆ strain (Fig. 6c). Thus, Sir3-mediated telomere clustering but not silencing is required for the maintenance of the quiescent state.

## Discussion

We report that the organization of the budding yeast genome changes drastically depending on the metabolic status of the cell. In particular, quiescent cells that sustain long-term viability or increased CLS form a discrete subcompartment of telomeric silent chromatin in the most internal part of the nucleus (Fig. 6d).

### Dynamics of nuclear organization upon carbon source exhaustion

We describe the dynamics of nuclear organization upon two major metabolic transitions: from fermentation to respiration and from respiration to SP. First, we show that telomere clusters, which are known to form three to five foci at the nuclear periphery in cells undergoing fermentation, form brighter and fewer foci after the diauxic shift. Furthermore, when cells exhaust the carbon source after respiration and enter the SP, these foci further group into a hypercluster located in the center of the nucleus in SP cells able to sustain long-term viability.

### SIR-mediated telomere clustering drives chromosome conformation in long-lived quiescent cells

Genomic 3C analyses reveal that long-lived SP cells display increased constraints in their nuclear architecture, which appears to be driven by the clustering of telomeres. Because *S. cerevisiae* chromosomes exhibit such differences in size, mechanical constraints are likely to play significant roles on the organization of chromosomes tethered at both their centromere and telomeric regions. The positioning of the cluster in the middle of the nuclear space may actually reflect this physical constraint imposed by the smallest chromosome arms. As *SIR3*-deleted cells are unable to form telomere hyperclusters in quiescence and show a global organization that is similar to that of G1 cells, we conclude that SIR-mediated telomere clustering drives the global reorganization of chromosomes in long-lived quiescent cells. Although both Sir3 and Sir4 are required for telomere hyperclustering, gene silencing is not necessary for this event, as demonstrated by expressing a silencing defective version of Sir3 [22]. Furthermore, telomere hyperclustering in quiescent cells is not driven by an increase in Sir3 protein or an increase of Sir3 spreading. As Sir3 may bind nucleosomes in more than one conformation [35], it is possible that telomere clustering after the diauxic shift is driven by specific post-translational modifications that increase Sir3 clustering function.

### Mitochondrial ROS commit cells to form telomere hyperclusters upon starvation

Importantly, we show that increased telomere clustering is not a general feature of cell cycle arrest, as it is observed only in quiescent cells able to sustain long-term viability. Furthermore, the ability to form telomere hyperclusters required mitochondrial activity and is acquired post-diauxic shift in the quiescent fraction of cells shown to have a six-fold higher respiration rate compared with the non-quiescent fraction of cells [36]. ROS, and more specifically H_2_O_2_ produced during respiration, are obvious candidates to trigger the commitment to form hyperclusters upon starvation [20]. Indeed, we show that mutants known to increase the cellular level of H_2_O_2_ form hyperclusters with a higher rate and faster kinetics than wild-type cells. Furthermore, treating pre-diauxic shift cells with a sub-lethal dose of H_2_O_2_ is sufficient to commit cells to form telomere hyperclusters upon starvation and to sustain long-term viability. This commitment could be mediated by the checkpoint kinase Rad53, which is activated at these levels of H_2_O_2_ [37], thus allowing crosstalk between mitochondria and the nucleus [38, 39].

### Potential benefits of telomere hyperclustering for CLS

Although alterations of nuclear architecture have been reported upon differentiation [40] and in quiescent metazoan cells [41], the function of this reorganization remains elusive. Interestingly, dramatic changes in the distribution of silent chromatin are observed in mammalian senescent cells with the formation of senescence-associated heterochromatin foci, which are thought to contribute to the stability of the cell cycle arrest [42]. Another striking example of genome reorganization comes from rod photoreceptor cells of nocturnal rodents. In these cells, the nuclei exhibit an “inverted organization” — that is, reminiscent to the hypercluster observed in long live yeast cells — probably as an adaptation to limited light [43].

The large reorganization of budding yeast telomeres into a hypercluster concomitant with an important metabolic adaptation most likely provides a survival advantage in the long-term. Accordingly, *sir3*∆ strains, which cannot form telomere clusters, show a modest reduction in longevity compared with wild-type strains, when SP cultures (after 3 days in rich medium) were shifted to water. This is consistent with the findings of [38]. However, quiescent cells purified from 7-day cultures of prototroph W303 strains showed no difference in the lifespan of *sir3*∆ or *sir4*∆ and wild-type cells (Linda Breeden, personal communication), possibly due to strain or experimental procedure variations. Importantly, the viability defect that we observed is rescued by expressing a *SIR3* allele that is competent for telomere clustering but defective for silencing (*sir3-A2Q* mutant [22]), indicating that telomere clustering in quiescence has a positive effect on CLS independent of gene silencing under our conditions.

We propose that telomere hyperclusters could influence survival by protecting telomeres from degradation, fusion, and/or ectopic recombination events. Alternatively, telomere hyperclustering in quiescence could also be a way to sequester multifunctional factors that could have deleterious effects if localized to nuclear subcompartments where they are not needed. Such a factor could be the sirtuin Sir2, since it plays a pro-aging role by regulating cytoplasmic enzymes involved in carbon metabolism [44, 45].

## Conclusions

 By establishing that the nuclear organization of quiescent cells significantly differs from the well-described organization of cells grown in nutrient-replete conditions, our study sets the ground to (re)interpret studies on nuclear processes in the context of quiescence and aging. Moreover, our results unravel a novel connection between nuclear organization and aging, paving the way for future experiments analyzing the importance of nuclear organization for chronological lifespan.

## Materials and methods

### Media and growth conditions

All yeast strains used in this work are listed in Additional file 7 and are from the W303 background [46] except for the strains used for the HiC experiment (BY4741 ). Gene deletions and gene tagging were performed by PCR-based gene targeting [46, 47].

Yeast cells were grown in rich medium (YPD, yeast extract-peptone-dextrose) at 30 °C.

Induction of quiescence by carbon source exhaustion was performed as follows. Yeast cells were inoculated in YPD and grown overnight. The following day, cultures were diluted to an optical density of 0.2 (OD_600nm_) and grown at 30 °C in agitation for 5–6 h (fermentation), 24–48 h (respiration) or more than 7 days (SP). Levels of glucose in the medium were determined by using the D-Glucose HK assay kit (Megazyme). Induction of quiescence by carbon source starvation was performed by growing the cells in YPD at 30 °C (before or after glucose exhaustion) and then transferring them to exhausted YPD or sterile water for at least 16 h. For nitrogen starvation experiments, cells were grown to an OD_600nm_ of 1 and transferred to a synthetic medium containing 0.17 % yeast nitrogen base (MP Biomedical) and 2 % glucose.

### Density gradient fractionation

For density gradient fractionation, a solution of Percoll (Sigma-Aldrich) with a final NaCl concentration of 167 mM was added to a 30 ml Corex tube and centrifuged at 13,000 rpm for 20 min.

Approximately 2 × 10^9^ cells were harvested, resuspended in 1 ml Tris buffer, added to the preformed gradient and centrifuged at 400 g_av_ for 60 min at 20 °C. Density gradient tubes were imaged, and fractions collected, washed once in water, and used directly for assays or split into aliquots, pelleted, and frozen in liquid nitrogen. Cell number was determined for each fraction.

### Viability (colony forming unit) assay

To test quiescent cells’ colony forming ability, cultures were grown as indicated. After 24 h of quiescence induction (day 1 CLS), 50 μl of each culture was collected, diluted 1:1.2 × 10^6^ and plated in YPD plates. Culture tubes were agitated at 30 °C for 7 days and plated. Colonies were counted after 3 days at 30 °C. Day 7 CLS was normalized to day 1 CLS. Plots represent the mean value obtained for at least three independent experiments; error bars correspond to standard error of the mean.

### H_2_O_2_ treatment

To test whether direct addition of ROS in the medium of cultures undergoing fermentation could commit nuclei to form telomere hyperclusters during starvation, cells grown overnight were diluted to 0.002 OD_600nm_/ml in fresh YPD containing no drugs or H_2_O_2_ 1 mM, grown until they reached 1 OD_600nm_/ml, and then starved in water for at least 24 h.

### Protein immunoblotting

For protein isolation, 200 μl of trichloroacetic acid (TCA) 20 %, 200 μl of TCA buffer [20 mM Tris–HCl pH 8, 50 mM ammonium acetate, 2 mM EDTA, 1 mM phenylmethylsulfonyl fluoride (PMSF)], 1 μl of Protease inhibitor cocktail (Sigma-Aldrich), and 400 μl of acid-washed glass beads (710–1180 μm; Sigma-Aldrich) were added to 1 × 10^8^ pelleted cells. Cells were then disrupted by vigorous vortexing (1 min, two times). Resulting extracts were centrifuged for 30 min at 4 °C at 14,000 rpm, and pellets were resuspended in 200 μl of TCA-Laemmli loading buffer (120 mM Tris base, 3.5 % sodium dodecyl sulfate (SDS), 8 mM EDTA, 5 % β-mercaptoethanol, 1 mM PMSF, 15 % glycerol, 0.01 % bromophenol blue). Samples were boiled for 10 min and centrifuged at 14,000 rpm for 10 min. Aliquots were immediately loaded or frozen. For immunoblotting, we used custom-made polyclonal antibodies against Rap1 (Agrobio, raised against Rap1[358–828] recombinant protein (a generous gift from M.H. LeDu, CEA Saclay) and Sir3 at 1:5000 [22]. Loading was normalized according to H2A at 1:5000 (Abcam).

### Immuno-FISH

Immuno-FISH experiments were performed as in [22] with minor modifications. For quiescent cells, spheroplasting time was increased (20 min instead of 10 min).

### Microscopy

Sets of images from any given figure panel were acquired the same day using identical acquisition parameters, except for time course experiments where the same culture was imaged at different time points, using identical acquisition parameters and using a wild-type growing culture as control. Details are provided in Additional file 8.

### Quantification of Rap1 foci

A dedicated tool has been designed to find and quantify the telomere cluster in the 3D images acquired with fluorescence microscopy. Details are provided in Additional file 8.

### Construction of 3C libraries and sequencing

*S. cerevisiae* G1 daughter cells (strain BY4741) were recovered from an exponentially growing population through an elutriation procedure [48]. Long-lived quiescent cells were recovered as described above. 3C libraries were generated as described [49] with minor changes in the protocol. Briefly, the cells were cross-linked for 20 minutes with fresh formaldehyde (3 % final concentration), pooled as aliquots of 3 × 10^9^ cells, and stored at −80 °C until use. Aliquots were thawed on ice and resuspended in 6 ml 1× *Dpn*II buffer (NEB). The cells were then split into four tubes and lysed using a Precellys grinder (3 cycles of 6500 rpm, 30 s ON/60 s OFF) and VK05 beads. The cells were incubated for 3 h with 50 units of restriction enzyme under agitation (*Dpn*II; NEB). The digestion mix was then diluted into ligation buffer and a ligation was performed at 16 °C for 4 h followed by a decrosslinking step consisting of an overnight incubation at 65 °C in the presence of 250 μg/ml proteinase K in 6.2 mM EDTA. DNA was then precipitated, resuspended in TE buffer, and treated with RNAse.

The resulting 3C libraries were sheared and processed into Illumina libraries using custom-made versions of the Illumina paired-end adapters (Paired-End DNA Sample Prep Kit, Illumina PE-930-1001). Fragments of sizes between 400 and 800 bp were purified using a PippinPrep apparatus (SAGE Science), PCR amplified, and paired-end sequenced on an Illumina platform (HiSeq2000; 2 × 100 bp).

### Processing of paired-end reads

The raw data from each 3C experiment were processed as follows First, PCR duplicates were collapsed using the six Ns present on each of the custom-made adapters. Reads were then aligned using Bowtie 2 in its most sensitive mode against the *S. cerevisiae* reference genome [50]. Paired-end reads were aligned as follows: for each read the length of the sequence mapped was increased gradually from 20 bp until the mapping became unambiguous (mapping quality >40). Paired reads were aligned independently.

### Generation of contact maps

Each mapped read was assigned to a restriction fragment. Genome-wide contact matrices were built by binning the genome into units of 20 restriction fragments, resulting in 1797 × 1797 contact maps. The contact maps were subsequently filtered and normalized using the sequential component normalization procedure described in [51]. This procedure ensures that the sum over the column and lines of the matrix equals 1 and reduces the influence of biases inherent to the protocol. Full resolution contact maps binned at ten restriction fragments are available in the supplemental material section (Additional files 9, 10, 11 and 12). The 3D structures were directly computed from the normalized contact maps using ShRec3D [27]. The algorithm first computes a distance matrix from the contact map by assuming that the distance between each pair of beads is equal to the shortest path on the weighted graph associated with the inverse of the contact frequency between the two corresponding nodes. Multi-dimensional scaling is then applied to recover the optimal 3D coordinates from this distance matrix. To allow direct comparison between the structures obtained in different conditions we first re-scaled them to equalize the volume occupied by their associated convex hull. We then scaled the distances in each structure to account for the measured difference in size between nuclei in G0 and G1 daughter cells (1.5 and 1.7 μm, respectively; data not shown and [52]). Telomere pair distances were then directly computed from the structures to assess telomere re-organization.

### Data availability

The sequences of the chromosome conformation capture experiments reported in this paper have been deposited in BioProject with accession number PRJNA291473 [53]. Microarray data are available from the Gene Expression Omnibus (GEO) under the accession number [GEO:GSE71273]. Microscopy data are available from Figshare [54].

## Additional files

Additional file 1: Figure S1. Characterization of the SP silent chromatin hypercluster. **a** Western blot against Rap1 on crude extracts from exponential, respiratory, or stationary cultures of a WT strain (yAT1684). H2A antibody was used for the loading control. **b** Representative fluorescent images of wild-type (WT) strains tagged with Rap1-GFP “yAT 1684”, GFP-Sir2 “yAT405”, Sir3-GFP “yAT779” and GFP-Sir4 “yAT431” strains. Overnight liquid cultures were diluted to 0.2 OD_600nm_/ml and images were acquired after 5 h (1 OD_600nm_/ml, fermentation phase) and 7 days (40 OD_600nm_/ml, stationary phase). **c** Representative fluorescent image of a Rap1-GFP Sir3-mCherry-tagged strain “yAT194” from stationary phase cultures. We note that Sir3 associates with both telomeres and the rDNA in stationary phase cells. **d** Representative fluorescent images of Rap1-GFP in stationary cultures of WT “yAT1684” and *sir4*∆ “yAT2092” strains. **e** Representative fluorescent images of the nucleolar protein Sik1 tagged with mCherry during fermentation, respiration, and stationary phase (“yAT340”). **f** Representative fluorescent image of Rap1-GFP Dad2-mRFP (Duo1 And Dam1 interacting, an essential component of the microtubule–kinetochore interface) tagged stationary phase cells (“yAT2279”). **g** Representative fluorescent image of Sir3-mCherry Cse4-GFP-tagged strain “yAT2280” from stationary phase. Scale bar is 1 μm. (PDF 1343 kb) Additional file 2: Movie S1. Related to Fig. 3. Animated 3D reconstruction of the entire contact map of G1 cells. Each chromosome is represented as a chain of beads (1 bead = 20 kb), and the color code reflects the chromosome arm length, from *blue* for short arms to *red* for long arms. Each chromosome carries a *black bead* that corresponds to the centromere position. *Yellow beads* = subtelomeric regions; *black beads* = centromeres; *purple beads* = boundaries of the rDNA cluster on chromosome XII (in *pink*/*red*). (GIF 15597 kb) Additional file 3: Figure S2. SIR-mediated telomere clustering drives chromosome conformation in the dense fraction of SP cells. **a** Mean contacts frequencies between 100-kb centromeres windows in G1 *(blue)* and G0 quiescent cells *(red)*. Black and green curves: contacts between 100-kb segments randomly sampled in both conditions, to illustrate the absence of coverage biases after normalization. **b **Chromosome organization of WT and *sir3∆ *quiescent cells (the cryptic mating type locus *HML* was deleted to prevent pseudo-diploid effect). ii) Normalized contact matrix obtained for *hml∆* (left) *and *hml∆ sir3∆ (right) *cells. Color scale: contact frequencies from rare *(white)* to frequent *(dark blue)*. Red arrowheads: centromeres contacts; green and yellow arrowheads: telomere–telomere contacts in *hml∆ *and *hml∆ sir3∆* G0 cells, respectively. The 3D representations of the *hml∆ *and *hml∆ sir3∆* matrices are represented next to the contact maps. Each chromosome is represented as a chain of beads (1 bead = 20 kb), with color code reflecting the chromosome arm lengths, from short *(blue)* to long *(red)* arms. Yellow beads: subtelomeric regions; black beads: centromeres; purple beads: boundaries of the rDNA cluster. **c** Contact maps of W303 strain during exponentially growth (EXPO, left) and quiescence (G0, *right*). Red arrowheads: centromere clustering; green and yellow arrowheads: telomere–telomere contacts of two chromosomes (XIII and XV) in expo and G0 cells, respectively. Because of the low sequencing coverage and quality, the signal is not as strong as for data in Fig. 3 and the bins are larger (1 vector: 80 *DpnII *RFs). **d** Quantification of colocalization of 30-kb telomeric regions *(red dots) *compared with the distribution of the colocalization scores (box plot, two standard deviations) computed for 1000 random sets of 32 windows of 30 kb in the genome (excluding centromeric regions). The colocalization score is normalized by the sequencing depth for each dataset. Additional file 4: Movie S2. Related to Fig. 3. Animated 3D reconstruction of the entire contact map of long-lived SP cells (isolated from a SP culture by density gradient). Same annotations as in Additional file 2. (GIF 12057 kb) Additional file 5: Figure S3. Telomere hyperclustering is not due to slow growth. **a** Representative fluorescent image of Rap1-GFP tagged strain grown either at 30 °C or 25 °C in exponential phase (*top*) and then starved for 16 h in water before imaging (*bottom*). **b** Calcofluor staining of LD and HD fractions of a post DS culture after gradient separation. **c** Heat shock (HS) assay on the LD and HD fractions used in **b**. (PDF 11591 kb) Additional file 6: Figure S4. Mechanism driving telomere clustering in long-lived SP cells. **a** Western blot against Sir3 and H2A on crude extracts from exponential, respiratory, or stationary cultures of a wild-type (WT) strain (yAT1684). **b** Sir3 spreading at yeast subtelomeres in cells from an exponentially growing culture (*Fermentation*) or in cells isolated from the dense fraction of a SP culture (*Stationary HD*). ChIP-chip profiles (Sir3 enrichment Z score) correspond to the mean of two independent experiments. Pearson correlation between conditions is 0.95. Sir3 spreading at TELVIR was confirmed in independent experiments by ChIP-quantitative PCR for both conditions (not shown). Each panel spans the first 30 kb from each telomere and the heading color for each panel indicates the middle repeat element content of the corresponding telomere: Y’ XCR XCS (*beige*), Y’ XCS (*green*), XCS (*red*), or XCR XCS (*blue*). Each dot represents a data point and lines are drawn for visual purposes. **c** Quiescent *sir3*∆ cells are as thermotolerant as quiescent WT cells to heat shock (HS). Dilution assays are shown (starting at DO_600nm_ = 5 and diluted 1/5 each time). *Left*: growth control of exponential cells or 24 h LD cells. *Middle*: sensitivity to HS of WT exponential cells, 24 h LD cells or 24 h HD cells. *Right*: sensitivity to HS of WT or *sir3*∆ LD cells. **d** Stationary WT, *sir3*∆, *sir3-A2Q* cells are resistant to HS like WT cells. Dilution assays are shown (starting at DO_600nm_ = 1 and diluted 1/5 each time). *Left*: growth control. *Middle*: 30 min 52 °C HS. *Left*: 1 h 52 °C HS. **e** Stationary WT and *sir3*∆ cells that spent 14 days in water after glucose exhaustion show the same extent of thermotolerance to a 1 h 52 °C HS. Dilution assays are shown (starting at DO_600nm_ = 1 and diluted 1/5 each time). (PDF 12384 kb) Additional file 7: Table S1. Strains used in this study. (DOCX 22 kb) Additional file 8:
**Additional experimental procedures.** (DOCX 32 kb). Additional file 9:
**Full resolution contact map binned at 10 RF of G1 population presented in Fig.** 3 **.** (DAT 24.7 mb) Additional file 10:
**Full resolution contact map binned at 10 RF of G0 population presented in Fig.** 3 **.** (DAT 16.3 mb) Additional file 11:
**Full resolution contact map binned at 10 RF of G0 WT population presented in Additional file** 3 **.** (DAT 24.6 mb) Additional file 12:
**Full resolution contact map binned at 10 RF of G0** ***sir3Δ*** **population presented in Additional file** 3 **.** (DAT 24.6 mb)

## Abbreviations

3C: capture of chromosome conformation
3D: three-dimensional
bp: base pair
ChIP: chromatin immunoprecipitation
CLS: chronological lifespan
FISH: fluorescence in situ hybridization
GFP: green fluorescent protein
HD: high density
LD: low density
PMSF: phenylmethylsulfonyl fluoride
ROS: reactive oxygen species
SP: stationary phase
TCA: trichloroacetic acid
