# Supplementary figures and images for: Spatial reorganization of telomeres in long-lived quiescent cells
Source: Genome Biol. 2015 Sep 23;16(1):206. doi: 10.1186/s13059-015-0766-2 (PMC4581094; doi:10.1186/s13059-015-0766-2)

**A**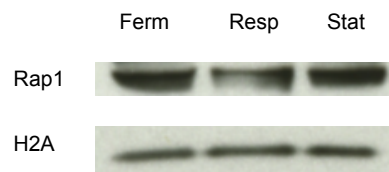**B**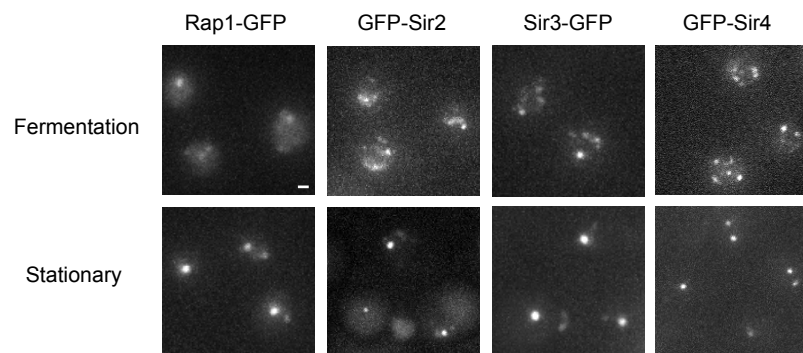**C**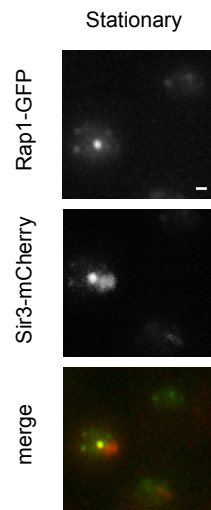**D**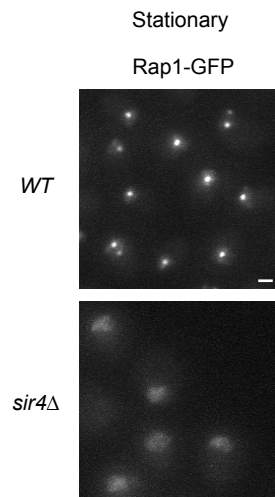**E**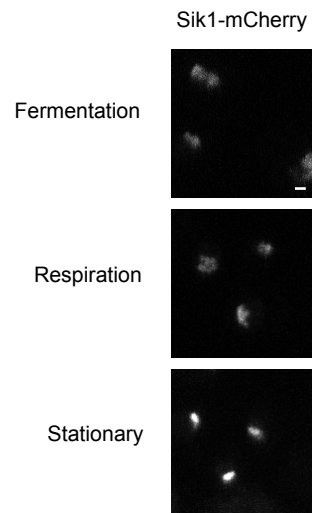**F**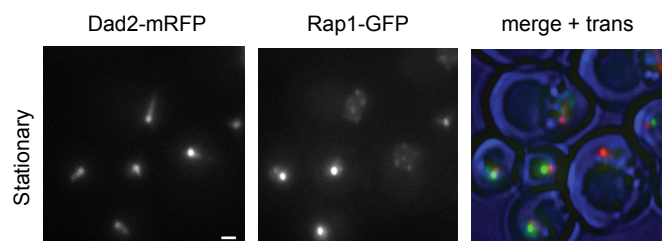**G**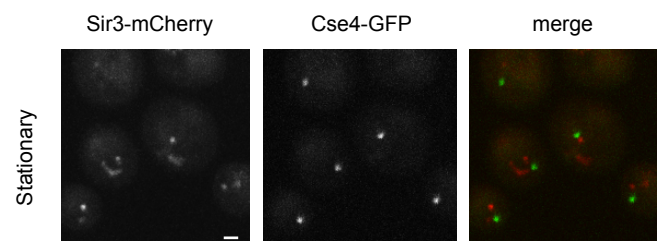

Supplement: Additional file 1: Figure S1. — Characterization of the SP silent chromatin hypercluster. a Western blot against Rap1 on crude extracts from exponential, respiratory, or stationary cultures of a WT strain (yAT1684). H2A antibody was used for the loading control. b Representative fluorescent images of wild-type (WT) strains tagged with Rap1-GFP “yAT 1684”, GFP-Sir2 “yAT405”, Sir3-GFP “yAT779” and GFP-Sir4 “yAT431” strains. Overnight liquid cultures were diluted to 0.2 OD600nm/ml and images were acquired after 5 h (1 OD600nm/ml, fermentation phase) and 7 days (40 OD600nm/ml, stationary phase). c Representative fluorescent image of a Rap1-GFP Sir3-mCherry-tagged strain “yAT194” from stationary phase cultures. We note that Sir3 associates with both telomeres and the rDNA in stationary phase cells. d Representative fluorescent images of Rap1-GFP in stationary cultures of WT “yAT1684” and sir4∆ “yAT2092” strains. e Representative fluorescent images of the nucleolar protein Sik1 tagged with mCherry during fermentation, respiration, and stationary phase (“yAT340”). f Representative fluorescent image of Rap1-GFP Dad2-mRFP (Duo1 And Dam1 interacting, an essential component of the microtubule–kinetochore interface) tagged stationary phase cells (“yAT2279”). g Representative fluorescent image of Sir3-mCherry Cse4-GFP-tagged strain “yAT2280” from stationary phase. Scale bar is 1 μm. (PDF 1343 kb) [file 13059_2015_766_MOESM1_ESM.pdf]

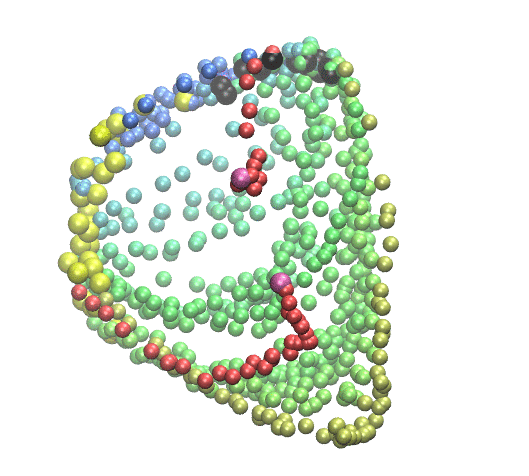

Supplement: Additional file 2: Movie S1. — Related to Fig. 3. Animated 3D reconstruction of the entire contact map of G1 cells. Each chromosome is represented as a chain of beads (1 bead = 20 kb), and the color code reflects the chromosome arm length, from blue for short arms to red for long arms. Each chromosome carries a black bead that corresponds to the centromere position. Yellow beads = subtelomeric regions; black beads = centromeres; purple beads = boundaries of the rDNA cluster on chromosome XII (in pink/red). (GIF 15597 kb) [file 13059_2015_766_MOESM2_ESM.gif]

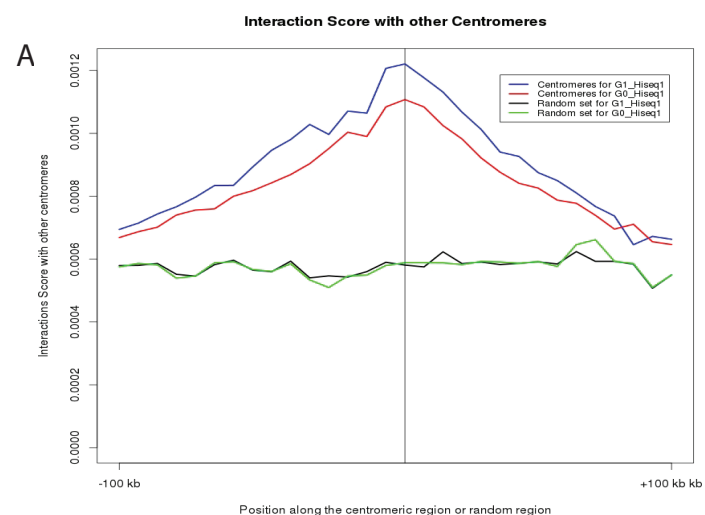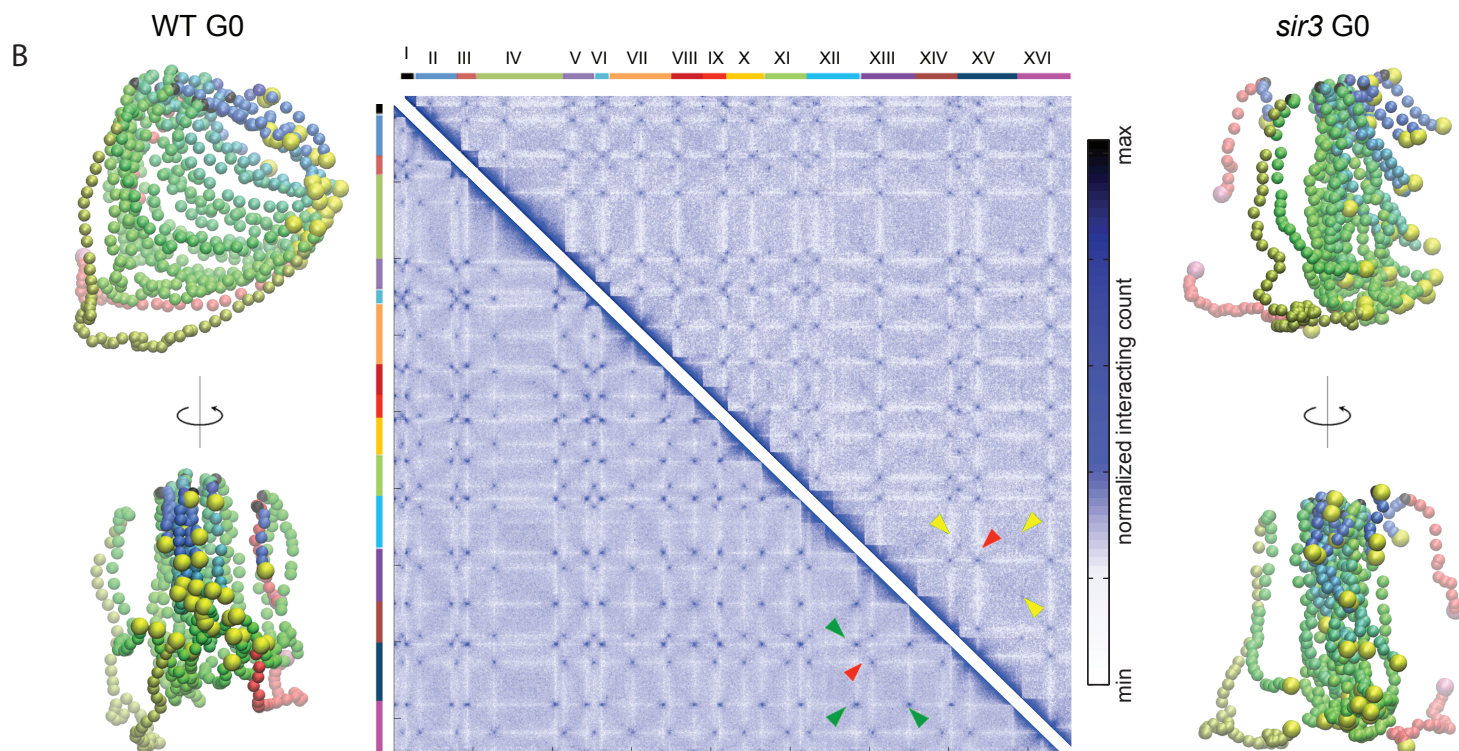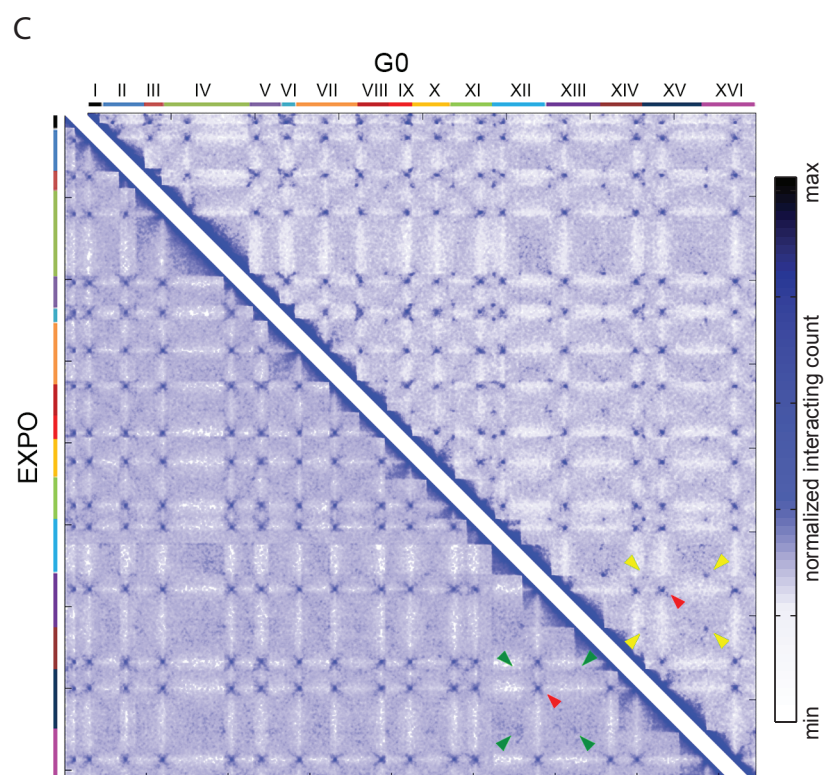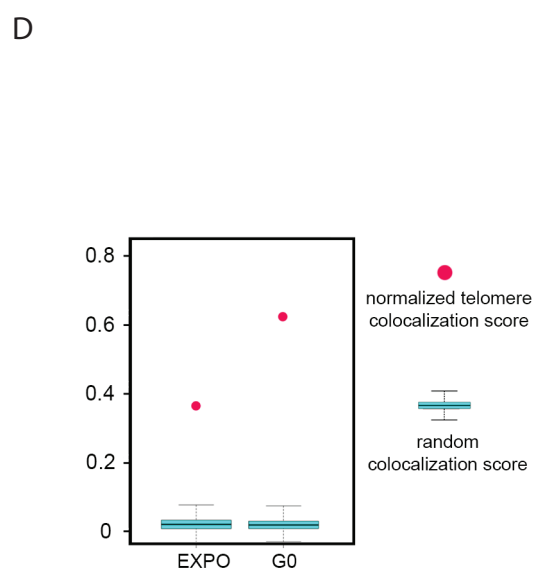

Figure S2 Guidi et al.

Supplement: Additional file 3: Figure S2. — SIR-mediated telomere clustering drives chromosome conformation in the dense fraction of SP cells. a Mean contacts frequencies between 100-kb centromeres windows in G1 (blue) and G0 quiescent cells (red). Black and green curves: contacts between 100-kb segments randomly sampled in both conditions, to illustrate the absence of coverage biases after normalization. b Chromosome organization of WT and sir3∆ quiescent cells (the cryptic mating type locus HML was deleted to prevent pseudo-diploid effect). ii) Normalized contact matrix obtained for hml∆* (left) and hml∆ sir3∆ (right) cells. Color scale: contact frequencies from rare (white) to frequent (dark blue). Red arrowheads: centromeres contacts; green and yellow arrowheads: telomere–telomere contacts in hml∆ and hml∆ sir3∆ G0 cells, respectively. The 3D representations of the hml∆ and hml∆ sir3∆ matrices are represented next to the contact maps. Each chromosome is represented as a chain of beads (1 bead = 20 kb), with color code reflecting the chromosome arm lengths, from short (blue) to long (red) arms. Yellow beads: subtelomeric regions; black beads: centromeres; purple beads: boundaries of the rDNA cluster. c Contact maps of W303 strain during exponentially growth (EXPO, left) and quiescence (G0, right). Red arrowheads: centromere clustering; green and yellow arrowheads: telomere–telomere contacts of two chromosomes (XIII and XV) in expo and G0 cells, respectively. Because of the low sequencing coverage and quality, the signal is not as strong as for data in Fig. 3 and the bins are larger (1 vector: 80 DpnII RFs). d Quantification of colocalization of 30-kb telomeric regions (red dots) compared with the distribution of the colocalization scores (box plot, two standard deviations) computed for 1000 random sets of 32 windows of 30 kb in the genome (excluding centromeric regions). The colocalization score is normalized by the sequencing depth for each dataset. [file 13059_2015_766_MOESM3_ESM.pdf]

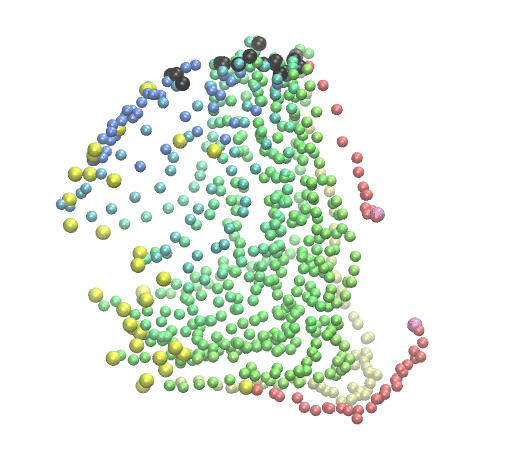

Supplement: Additional file 4: Movie S2. — Related to Fig. 3. Animated 3D reconstruction of the entire contact map of long-lived SP cells (isolated from a SP culture by density gradient). Same annotations as in Additional file 2. (GIF 12057 kb) [file 13059_2015_766_MOESM4_ESM.gif]

**A**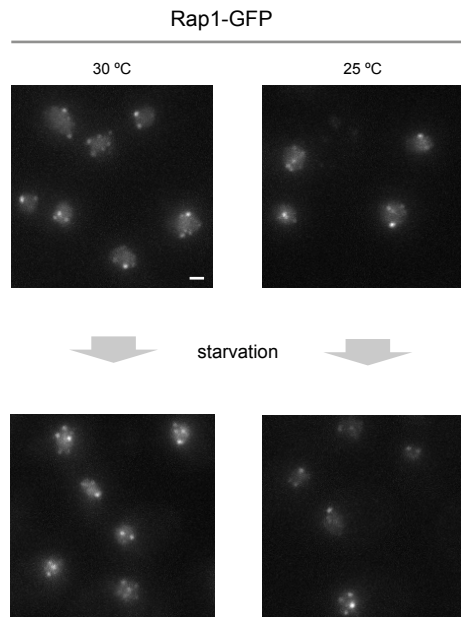**B**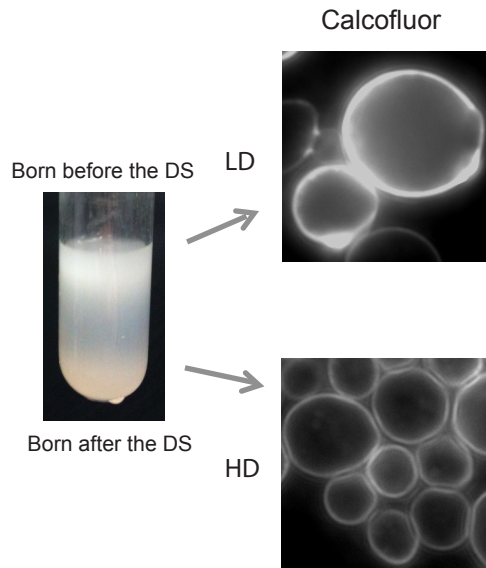**C**

Heat-shock

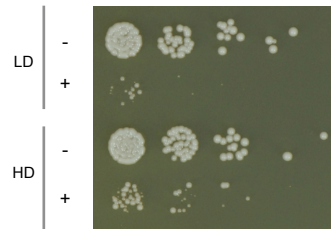

Supplement: Additional file 5: Figure S3. — Telomere hyperclustering is not due to slow growth. a Representative fluorescent image of Rap1-GFP tagged strain grown either at 30 °C or 25 °C in exponential phase (top) and then starved for 16 h in water before imaging (bottom). b Calcofluor staining of LD and HD fractions of a post DS culture after gradient separation. c Heat shock (HS) assay on the LD and HD fractions used in b. (PDF 11591 kb) [file 13059_2015_766_MOESM5_ESM.pdf]

**A**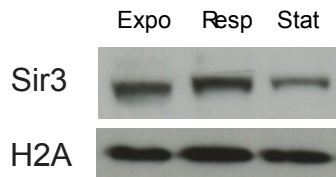**B**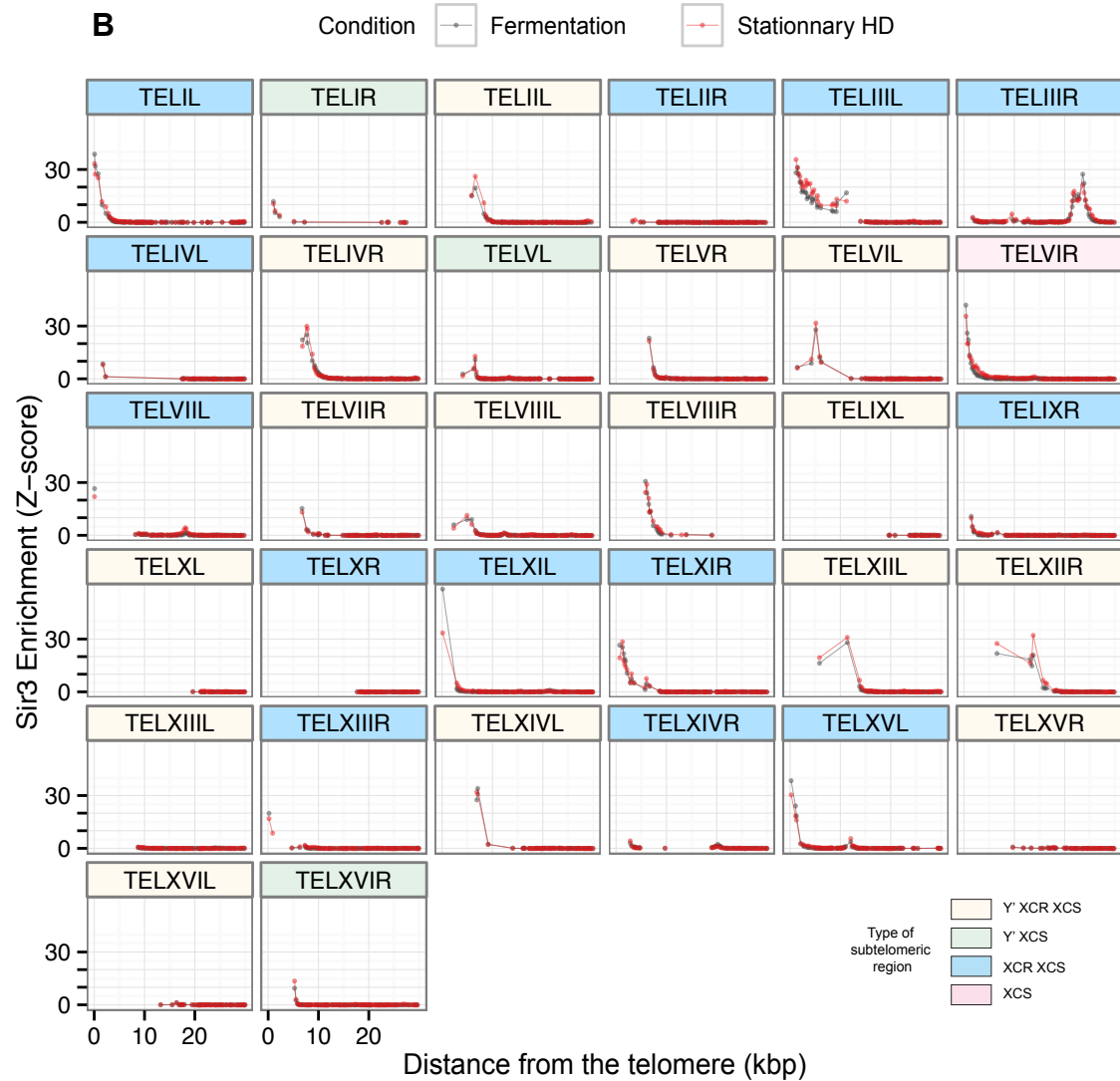**C**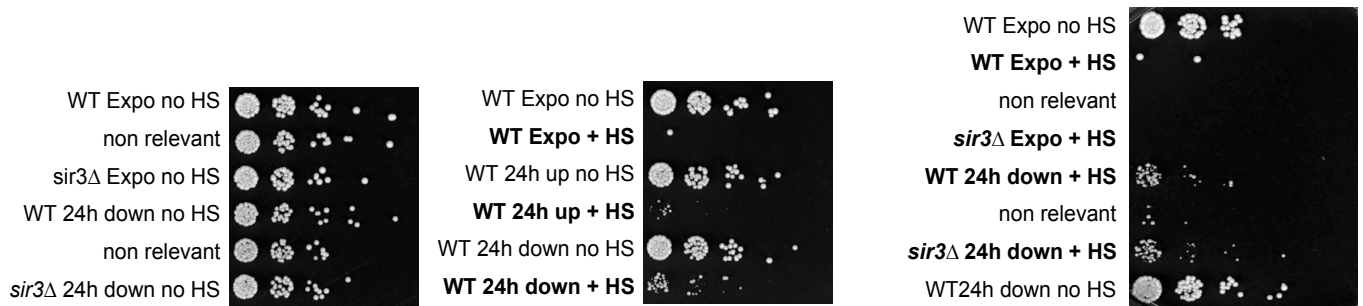**D**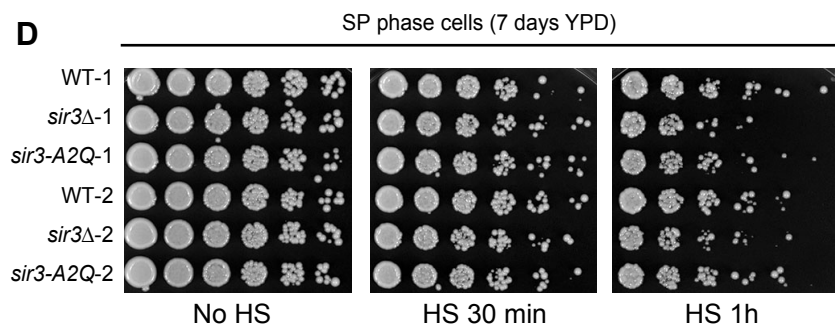**E**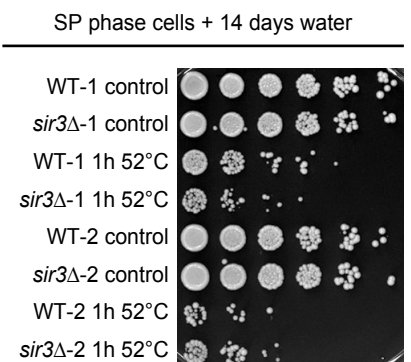

Supplement: Additional file 6: Figure S4. — Mechanism driving telomere clustering in long-lived SP cells. a Western blot against Sir3 and H2A on crude extracts from exponential, respiratory, or stationary cultures of a wild-type (WT) strain (yAT1684). b Sir3 spreading at yeast subtelomeres in cells from an exponentially growing culture (Fermentation) or in cells isolated from the dense fraction of a SP culture (Stationary HD). ChIP-chip profiles (Sir3 enrichment Z score) correspond to the mean of two independent experiments. Pearson correlation between conditions is 0.95. Sir3 spreading at TELVIR was confirmed in independent experiments by ChIP-quantitative PCR for both conditions (not shown). Each panel spans the first 30 kb from each telomere and the heading color for each panel indicates the middle repeat element content of the corresponding telomere: Y’ XCR XCS (beige), Y’ XCS (green), XCS (red), or XCR XCS (blue). Each dot represents a data point and lines are drawn for visual purposes. c Quiescent sir3∆ cells are as thermotolerant as quiescent WT cells to heat shock (HS). Dilution assays are shown (starting at DO600nm = 5 and diluted 1/5 each time). Left: growth control of exponential cells or 24 h LD cells. Middle: sensitivity to HS of WT exponential cells, 24 h LD cells or 24 h HD cells. Right: sensitivity to HS of WT or sir3∆ LD cells. d Stationary WT, sir3∆, sir3-A2Q cells are resistant to HS like WT cells. Dilution assays are shown (starting at DO600nm = 1 and diluted 1/5 each time). Left: growth control. Middle: 30 min 52 °C HS. Left: 1 h 52 °C HS. e Stationary WT and sir3∆ cells that spent 14 days in water after glucose exhaustion show the same extent of thermotolerance to a 1 h 52 °C HS. Dilution assays are shown (starting at DO600nm = 1 and diluted 1/5 each time). (PDF 12384 kb) [file 13059_2015_766_MOESM6_ESM.pdf]
