# Supplementary material for: Spatial reorganization of telomeres in long-lived quiescent cells
Source: Genome Biol. 2015 Sep 23;16(1):206. doi: 10.1186/s13059-015-0766-2 (PMC4581094; doi:10.1186/s13059-015-0766-2)
Supplement: Additional file 7: Table S1. — Strains used in this study. (DOCX 22 kb) [file 13059_2015_766_MOESM7_ESM.docx]

| **Additional file 7: Table S1: Strains used in this study** | |
| --- | --- |
| W303:  yAT194 | ade2-1 can1-100 his3-11,15 leu2-3,112 rad5- trp1-1 ura3-1  MATα adh4::URA3-4xUASG-(C1-3A)n ppr1∆::HIS3 rap1::GFP-RAP1(LEU2) sir3::SIR3-mcherry::kan(ADE2) |
| yAT340 | MATα ade2-1::ADE2 sik1::SIK1-mRFP(KanMX) rap1::GFP-RAP1(LEU2) |
| yAT405 | MATα ade2-1::ADE2 sik1::SIK1-mRFP(KanMX) sir2::SIR2-yeGFP(HPH) |
| yAT431 | MATa ade2-1::ADE2 sik1::SIK1-mRFP(KanMX) sir4::GFP-SIR4(URA3) |
| yAT779 | MATa ade2-1::ADE2 sir3::SIR3-GFP(LEU2) |
| yAT1684 | MATa hml∆::HPH RAD5 rap1::GFP-RAP1(LEU2) RDN1::ADE2 |
| yAT2022 | MATa hml∆::HPH RAD5 rap1::GFP-RAP1(LEU2) RDN1::ADE2 sir3∆::KanMX |
| yAT2092 | MATa hml∆::HPH RAD5 rap1::GFP-RAP1(LEU2) RDN1::ADE2 sir4∆::HIS5 |
| yAT2279 | MATa dad2::DAD2-TagRFP-T(SpHIS5) hml∆::HPH rap1::GFP-RAP1(LEU2) RDN1::ADE2 |
| yAT2280 | MATα cse4::CSE4-GFP(S65T) RAD5 sir3::SIR3-mcherry::kan(ADE2) |
| yAT2332 | MATa hml∆::HPH RAD5 rap1::GFP-RAP1(LEU2) RDN1::ADE2 sir3∆::KanMX pSIR3::SIR3(HIS3) |
| yAT2333 | MATa hml∆::HPH RAD5 rap1::GFP-RAP1(LEU2) RDN1::ADE2 sir3∆::KanMX pSIR3::sir3-A2Q(HIS3) |
| yAT2338 | MATa his3::HIS3 hml∆::HPH RAD5 rap1::GFP-RAP1(LEU2) RDN1::ADE2 sir3∆::KanMX |
| yAT2407 | MATa ade2-1::ADE2 hml∆::HPH nup49::GFP-NUP49 rap1::yemRFP-RAP1(LEU2) |
| yAT2543 | MATa ctt1∆::HPH hml∆::HPH RAD5 rap1::GFP-RAP1(LEU2) RDN1::ADE2 |
| yAT2546  BY :  BY4141  yAT2527  yAT2540 | MATa hml∆::HPH RAD5 rap1::GFP-RAP1(LEU2) RDN1::ADE2 sod2::GPD-SOD2(NAT)  his3∆0 leu2∆0 met15∆0 ura3∆0  MATa  MATa hml∆::HPH  MATa hml∆::HPH sir3∆::KanMX |
