# Supplementary material for: Spatial reorganization of telomeres in long-lived quiescent cells
Source: Genome Biol. 2015 Sep 23;16(1):206. doi: 10.1186/s13059-015-0766-2 (PMC4581094; doi:10.1186/s13059-015-0766-2)
Supplement: Additional file 8: — Additional experimental procedures. (DOCX 32 kb). [file 13059_2015_766_MOESM8_ESM.docx]

**Additional Experimental Procedures:**

**Microscopy**

The live-cell images were acquired using a wide-field microscopy system based on an inverted microscope (TE2000; Nikon) equipped with a 100x/1.4 NA immersion objective, a charge-coupled device (CCD) camera (Coolsnap HQ2; Photometrics), a collimated white light-emitting diode for the transmission, and a UV filter on the two illumination paths (LP 400 and GG400; Nikon). A xenon arc lamp (Lambda LS; Sutter Instrument Co.) was used to illuminate the samples for all the figures except Figure 4. For Figure 4, the lamp used was a Spectra X light engine (Lumencor, Inc.). A dual-View micro-imager (Optical Insights) was positioned in the optical path. When used, this device spatially split emitted light and allowed the simultaneous measurement of two-color information on the same sensor. GFP-mCherry two-color images were acquired simultaneously on two halves of the same sensor using a GFP-mCherry filter block (excitation: double BP, 460–490/550–590 nm and dichroic double BP 500–550/ 600–665 nm) and the Dual View. The Dual View was equipped with adapted filter sets to observe green fluorescence (GFP, dichroic 565 nm and emission BP 499–529 nm; Semrock) on the left channel and red fluorescence (mCherry, dichroic 565 nm and emission BP 604–656 nm; Semrock) on the right channel. A homemade ImageJ macro (National Institutes of Health) was used to align and recombine channels. The position shift was estimated using the correlation function peak in transmitted light data (which is the same in the two channels) and used for fluorescent image alignment. The whole system is piloted by MetaMorph software (Molecular Devices).

**Quantification of Rap1-foci**

A dedicated tool has been designed to find and quantify the telomere cluster in the 3D images acquired with fluorescence microscopy. The image processing and data analysis are fully automatic, and required no user intervention, except to reject cells defined as abnormal in this study (i.e. dead, dividing…). The analysis workflow is a process with two major steps: foci are firstly detected and counted on each nuclei using a macro on Fiji [[1](#_ENREF_1)], then the sub-pixel localization and the intensity quantification of all foci are evaluated using a Matlab routine. To identify the nuclei, the diffuse Rap1-GFP fluorescence signal from the raw data was considered as a nucleoplasm staining, whereas the deconvolved images are required to detect the foci. Deconvolution was made using the Meinel algorithm in Metamorph (eight iterations; Σ = 0.8; frequency 3; MDS Analytical Technologies). A median filtering was applied on both 3D stacks. The nuclei are identified on a z-projection (sum) of the 3D images, on which a Laplacian of Gaussian (LoG) filter and a thresholding have been applied to reveal the nuclei. As a result, a binarized mask of the nuclei is obtained. To improve the detection of foci and decrease false detection, the nuclei mask is applied to the 3D acquisition after a background substraction (using Gaussian blur). The resulting stack is then LoG-filtered, binarized and the foci detection is done using the plugin "3D object counter" [[2](#_ENREF_2)] allowing to extract the position of the detected foci in the stack (x,y,z). Finally the foci quantification (position, radius, intensity) is obtained by fitting the fluorescence intensity of the deconvolved 3D-image in the vicinity on each detected foci according to the general equation:

I(x,y) = B0 + I0 exp[-( (( (x-x0)cos(theta) + (y-y0)sin(theta))/sigmaX)^2 + ( (-(x-x0)sin(theta) + (y-y0)cos(theta)) /sigmaY )^2 )]

where B0 is background level, I0 is the intensity of the foci, x0 and y0 are the positions of the foci, sigmaX and sigmaY are the sizes of the foci in the x- and y-direction and theta is the mean orientation of the foci. Finally, a graphical user interface has been developed in Matlab to discard abnormal cell from the analysis, and to gather results with same experimental conditions.

In Fig 1C, foci intensity and distribution are shown for each condition. In the following figures, results are summarized in pie charts where the percentages of cells showing hyperclusters are indicated. Hyperclusters were defined as Rap1-GFP foci with intensity higher than 95 % of wild-type foci upon fermentation conditions (figure 2 and 3). The same threshold was applied for all conditions presented on the same panel to define hyperclusters. When comparing different growth conditions of the same strain, the exponential phase was used to set the threshold. When comparing different strains on the same growth conditions, the threshold was set on the control strain in these conditions.

Pie charts represent in black the percentage of cells with telomere hyperclusters. For all the figures, except Figure 4, cells with hyperclusters were defined as cells containing 1 or 2 Rap1-GFP foci with intensity levels above 95% of foci in exponentially growing cells. For Figure 4, cells with hyperclusters were defined as cells containing 1 to 4 Rap1-GFP foci with intensity levels above 95% of foci in exponentially growing cells.

**Chromatin immunoprecipitation and micro-array hybridization**

A total of 20 O.D equivalent of *WT* “yAT1684” cells from fermentation (1 OD_600nm_/ml) or from the HD fraction of SP cells were fixed in 20 mL with 0.9 % formaldehyde for 15 min at 30°C, quenched with 0.125 M glycine and washed twice in cold TBS 1x pH 7.6. Pellets were suspended in 1mL TBS 1X, centrifuged and frozen in liquid nitrogen for -80°C storage. All following steps were done at 4°C unless indicated. Pellets were re-suspended in 500 µL of lysis buffer (0.01% SDS, 1.1% TritonX-100, 1.2 mM EDTA pH 8, 16.7 mM Tris pH8, 167 mM NaCl, 0.5 % BSA, 0.02 g.L^-1^ tRNA and 2.5 µL of protease inhibitor from SIGMA P1860) and mechanically lysed by three cycles of 30 s with 710-1.180 µm glass beads followed by two cycles of 30 s with 500 µm zirconium/silica beads (Biospec Products) using a Fastprep instrument (MP Biomedicals). Each bead beating cycle was followed by 5 min incubation on ice. The chromatin was fragmented to a mean size of 500 bp by sonication in the Bioruptor XL (Diagenode) for 14 min at high power with 30 s on / 30 s off and centrifuged 5 min at 13 000 rpm. 10 µL were kept to be used as Input DNA. Cleared lysate was incubated overnight with 1 µL of polyclonal antibody anti-Sir3 (Agro-bio). 50 µL of magnetic beads protein A (NEB) were added to the mixture and incubated for 4h at 4°C. Magnetic beads were washed sequentially with lysis buffer, twice with RIPA buffer (0.1% SDS, 10mM Tris pH7.6, 1mM EDTA pH8, 0,1% sodium deoxycholate and 1% TritonX-100), twice with RIPA buffer supplemented with 300 mM NaCl, twice in LiCl buffer (250 mM LiCl, 0.5% NP40, 0.5 % sodium deoxycholate), with TE 0.2% TritonX-100 and with TE. Input were diluted 10x with elution buffer (50mM Tris, 10mM EDTA pH8, 1%SDS) and beads were re-suspended in 100 µL elution buffer. A reversal cross-linking was performed by heating samples overnight at 65°C. Proteins were digested with proteinase K in presence of glycogen and the remaining DNA was purified on QIAquick PCR purification columns. Finally, samples were treated with 29 µg.mL^-1^ RNAse A 30 min at 37°C.

For microarray hybridization 4/5 of the immunoprecipitated DNA and 3/5 of the DNA from the input were ethanol precipitated and re-suspended in 10µL of water (Gibco). Purified material was amplified, incorporating amino-allyl-dUTP as described [[3](#_ENREF_3)]. The size of amplified fragments (~500 bp) was assessed by gel electrophoresis. For each sample 1-2 µg of amplified DNA was coupled either with Cy5 (immunoprecipitated sample) or Cy3 (input sample) and hybridized on 44k yeast whole genome tiling array (Agilent) as described in [[3](#_ENREF_3)]

**Microarray data acquisition, analysis and visualization**

Microarray was imaged using a Genepix 4000 scanner and quantified using GenePix Pro6.1 as described in [[3](#_ENREF_3)] with a minor modification: probes saturating in the Cy5 channel were kept in the downstream analysis. Enrichment scores for each replicate were computed by applying a z-score transformation using R scale function. Data visualization was done using the R package ggplot2. All scripts used are available upon request. Data have been submitted to Gene Expression Omnibus (GEO) under the accession GSE71273.

**Thermotolerance assays**

Cells were submitted to a 52°C heat shock (for 10, 20, 30 minutes or 1 hour) followed by 10 minutes on ice before plating.

**Supplemental references**

1. Schindelin J, Arganda-Carreras I, Frise E, Kaynig V, Longair M, Pietzsch T, Preibisch S, Rueden C, Saalfeld S, Schmid B, et al: **Fiji: an open-source platform for biological-image analysis.** *Nature methods* 2012, **9:**676-682.

2. Bolte S, Cordelieres FP: **A guided tour into subcellular colocalization analysis in light microscopy.** *Journal of microscopy* 2006, **224:**213-232.

3. Borde V, Robine N, Lin W, Bonfils S, Geli V, Nicolas A: **Histone H3 lysine 4 trimethylation marks meiotic recombination initiation sites.** *The EMBO journal* 2009, **28:**99-111.
